# Identification of the Immunogenic Spore and Vegetative Proteins of *Bacillus anthracis* Vaccine Strain A16R

**DOI:** 10.1371/journal.pone.0057959

**Published:** 2013-03-13

**Authors:** Xiankai Liu, Dongshu Wang, Jingxiao Ren, Chao Tong, Erling Feng, Xuefang Wang, Li Zhu, Hengliang Wang

**Affiliations:** State Key Laboratory of Pathogen and Biosecurity, Beijing Institute of Biotechnology, Beijing, People's Republic of China; Universidad Nacional de La Plata, Argentina

## Abstract

Immunoproteomics was used to screen the immunogenic spore and vegetative proteins of *Bacillus anthracis* vaccine strain A16R. The spore and vegetative proteins were separated by 2D gel electrophoresis and transferred to polyvinylidene difluoride membranes, and then western blotting was performed with rabbit immune serum against *B.anthracis* live spores. Immunogenic spots were cut and digested by trypsin. Matrix-assisted laser desorption ionization time-of-flight mass spectrometry was performed to identify the proteins. As a result, 11 and 45 immunogenic proteins were identified in the spores and vegetative cells, respectively; 26 of which have not been reported previously. To verify their immunogenicity, 12 of the identified proteins were selected to be expressed, and the immune sera from the mice vaccinated by the 12 expressed proteins, except BA0887, had a specific western blot band with the A16R whole cellular lytic proteins. Some of these immunogenic proteins might be used as novel vaccine candidates themselves or for enhancing the protective efficacy of a protective-antigen-based vaccine.

## Introduction

*Bacillus anthracis* is a Gram-positive bacterium, either aerobic or facultative anaerobic. It is the causative agent of anthrax, and has been reported as a potential bioterrorism weapon due to its dormant spores that can survive with high stability and low mortality in nutritive conditions and other severe environments[1]. Anthrax infection occurs via introduction of *B.anthracis* spores into a skin abrasion, inhalation, or ingestion [2]. Inhalation anthrax is the most severe form of the disease and is initiated by uptake of infective spores by alveolar macrophages. The ingested spores germinate into vegetative bacilli that invade the bloodstream, where they multiply massively and express toxins and virulence factors. If not treated by prompt antibiotic administration, the disease results in death of the infected organism, as a consequence of toxemia and bacteremia[2]. At the same time, the protective antigen (PA) and some other unknown immunogenic proteins of the *Bacillus anthracis* are able to elicit a humoral immune response in the course of bacterial infections.

Fully virulent forms of *B.anthracis* carry two large plasmids: pXO1 and pXO2, which are involved in toxin production and capsule formation, respectively. Several studies have shown that strains with only one plasmid are attenuated in animal hosts. The tripartite toxin, the protective antigen (PA), lethal factor (LF) and edema factor (EF), are encoded by the genes *pagA, lef*, and *cya* on pXO1, respectively. PA has no toxic effect itself, but it plays an essential role in recognizing and binding a membrane receptor on the surface of target cells [3], [4] and generating the portal that mediates the entry of EF and LF into the cells, where their detrimental effects occur. All of these are associated with *B.anthracis* pathogenicity.

Although early treatment with antibiotics such as penicillin, doxycycline or ciprofloxacin can halt progression of infection, vaccination remains the preferred method for prevention of infection and eradication of the causative agent. The currently used anthrax vaccines in the United States (Anthrax Vaccine Adsorbed; AVA) and United Kingdom (Anthrax Vaccine Precipitated; AVP) are component vaccines that are based on culture filtrates of avirulent *B.anthracis* strains lacking the pXO2 plasmid[5], [6]. These pXO1-containing strains produce large amounts of PA, which is the major component of each vaccine. Although the efficacy and safety of both vaccines have been established, there are concerns over side effects. These may be caused by residual enzymatic components in the filtrate, which combine with PA to form active toxin complexes[6]. Some recipients probably also develop hypersensitivity to unidentified components of the vaccine. It is possible that other factors in the vaccine including the aluminum adjuvant and preservatives may be responsible for adverse clinical symptoms. However, the Russian(STI-1) and Chinese (A16R; used in this study) anthrax vaccines utilize a live attenuated PA-producing form of *B.anthracis* spores that cannot produce capsule because of lose of the capsule-coding plasmid pXO2[7], [8]. Although the live spore anthrax vaccines have been reported to have greater efficacy than the AVA or AVP vaccine [9], due to their high reactogenicity, such live vaccines are suitable only for veterinary purposes in Western countries. Several studies have shown that this higher protective efficacy of the spore-base vaccine is due to the contribution of unidentified spore antigens that can augment the protective efficacy of PA-based vaccines [10], [11], [12]. Thus, it is rational that combining PA with one or some of the spore immunogenic proteins in a formulation may ultimately lead to a highly efficacious and safer vaccine.

Immunoproteomics is a science combining proteomics and immunology that facilitates screening many immunogenic proteins simultaneously[13], [14]. The spores are crucial to infection and persistence of *B.anthracis*, and the spores and vegetative cells can elicit a humoral immune response in the course of bacterial infections. Therefore we used the spore and the vegetative form of the *B.anthracis* vaccine strain A16R to identify immunogenic proteins that might augment the protective efficacy of PA-based vaccines. Our analysis revealed a list of conserved antigenic spore proteins and vegetative proteins of *B.anthracis* that may be potential candidates in the future for therapeutics and vaccines development. Some of these immunogenic proteins might be tested as novel vaccine candidates themselves or utilized for enhancing the protective efficacy of the vaccine available. Future work probing the identified immunogenic proteins with the serum of convalescent anthrax patients and *in vivo* protection experiments should be carried out to validate the immunogenicity and protective efficacy.

## Materials and Methods

### Bacterial strain and growth conditions

The attenuated vaccine strain A16R (pXO1^+^, pXO2^−^) of *B.anthracis* was used in this study. A16R vaccine was derived from a wild *B.anthracis* strain A16, which was isolated from a mule carcass that died of anthrax in the city of LangFang, Hebei province in 1953, by exposure to ultraviolet radiation, which resulted in lose of the capsule-coding plasmid pXO2. Cells were grown under the aerobic conditions in Brain Heart Infusion (BHI) or Luria–Bertani (LB) liquid medium at 37°C with vigorous agitation. *Escherichia coli* strains DH5α and BL21 used for prokaryotic expression were grown in LB agar or LB broth (Difco). Ampicillin was added at a concentration of 100 µg/ml.

### Preparation of live spores of *B.anthracis strain A16R*

The spores were isolated as described previously, with some modifications [15], [16]. *B.anthracis* was cultured first in BHI liquid medium overnight and then spread onto LB agar plates for several days until 95% of the spores formed. The spores were confirmed by spore staining and resuspended in distilled water. Spore suspensions were heated first at 45–48°C for 16–18 h to disrupt the vegetative cells, then 65°C for 1 h to kill any viable vegetative cells, washed extensively with ice-cold sterile 10% glycerol prepared in deionized water to remove the vegetative cells and debris, and resuspended in sterile deionized water. Gradient dilution was used to count the number of purified spores. The harvested spores were stored at −20°C for 1 week to 30 months.

### Preparation of rabbit immune serum against *B.anthracis* live spores

Serum preparation for 2D western blotting was as described previously [17]. Four New Zealand white rabbits were used. All animal experiments were approved by our Institution Animal Care and Use Committee (Beijing Institute of Biotechnology) and all experiments were done in accordance with the ethical guidelines of our Institution. Specific pathogen-free New Zealand white rabbits were purchased from Beijing Laboratory Animal Center (Beijing, China) and housed in pathogen free conditions. Serum was collected following multiple injections of live spores of *B.anthracis* A16R (pXO1^+^, pXO2^−^), gradually increasing the dosage from 10^5^ to 10^8^ CFU/ml, after 2 weeks for the first time, followed by 1-week intervals for the next three times. The first challenge was inguinal injection with 1 ml 10^5^ CFU/ml spores. The second challenge was intravenous injection with 1 ml 10^6^ CFU/ml spores. The third challenge was intravenous injection with 1 ml 10^7^ CFU/ml spores. The fourth challenge was subcutaneous injection with 1 ml 10^8^ CFU/ml spores. The sera were evaluated for the presence of antibody against total vegetative proteins by ELISA, using PA-D4 protein (domain 4 of PA, representing the 139 amino acids of the carboxyl terminal, which contains the host cell receptor binding site and the dominant protective epitodes of PA) as the coating antigen as described previously [18]. The antibody titer was 1∶12600.

### 2D gel electrophoresis (2-DE) and western blot analysis

#### Extraction of spore and vegetative proteins for 2-DE

The spore proteins extraction method was adapted from several previously reported protocols [19], [20]. Spores collected above were washed four times for 10 min at 8000 rpm with low-salt washing buffer (3 mM KCl, 1.5 mM KH_2_PO_4_, 68 mM NaCl, 9 mM NaH_2_PO_4_) and then resuspended in 1 ml lysis buffer containing ZOOM 2D protein solubilizing buffer-1 (Invitrogen, Carlsbad, CA, USA) and Protease Inhibitor Cocktail (Roche, Indianapolis, IN, USA). The samples were pulse sonicated for 10 min (pulse on 2 s, pulse off 6 s), followed by addition of the sample buffer (1 µl pH 3–10 IPG buffer and 1 U DNase-1 and 3 U RNase), incubated at room temperature for 30 min to eliminate DNA and RNA contamination, and centrifuged for 30 min at 20 000 rpm at 18°C to sediment the insoluble components. The supernatant was collected, and its protein concentration was determined using a PlusOne 2-D Quant Kit (GE Healthcare, Piscataway, NJ, USA), and 0.8-mg aliquots were stored at −70°C until use.

The vegetative cells of A16R were harvested when cultured for 23 h. Preparation of the whole cellular protein was performed as described previously [21]. Cells were grown aerobically in 50 ml BHI broth at 37°C for 23 h. Cells were harvested and centrifuged for 10 min at 8000 rpm (Sigma 3K12, St Louis, MO, USA) at 4°C, and the pellet was washed four times for 10 min at 8000 rpm with low-salt washing buffer. The pellet was resuspended in 5 ml lysis buffer containing ZOOM 2D protein solubilizing buffer-1 (Invitrogen) and Protease Inhibitor Cocktail (Roche), and ruptured by sonication for 15 min at 0°C. After adding 2.5 mg RNaseA, 100 U RQ1 DNase and 50 µl IPG buffer (pH 3–10), the lysed cell suspension was kept at room temperature for 1 h to solubilize proteins efficiently, and centrifuged for 20 min at 20 000 rpm to sediment the insoluble components. The supernatant was collected, and its protein concentration was determined using a PlusOne 2-D Quant Kit (GE Healthcare), and 0.8-mg aliquots were stored at −70°C until use.

#### 2-DE separation of spore and vegetative proteins

To achieve better separation, pH 4–7, 3.9–5.1, 4.7–5.9 and 5–6 Immobilized pH Gradient (IPG) strips (18 cm; GE Healthcare) were used in the isoelectric focusing analysis. One aliquot (0.8 mg) protein sample was treated first with the 2-D Clean-Up kit (GE Healthcare), and then resuspended in 350 µl rehydration buffer [7 M urea, 2 M thiourea, 4% (w/v) CHAPS, 50 mM DTT, 0.5% IPG Buffer (same pH range of the IPG strip)]. Proteins were subjected to proteome analysis by 2-DE as described previously, with modifications[21], [22]. The sample was used to rehydrate an 18-cm IPG strip for 12 h at 20°C. The following focusing parameters were applied: 1 h 300 V; 1 h 600 V; 1 h 1000 V; 1 h 8000 V; followed by the 8000 V holding step at the end of the run (64000 V·h total). After focusing was completed, IPG strips were equilibrated with 1% (w/v) DTT in equilibration base buffer [50 mM Tris–HCl (pH 8.8), 6 M urea, 30% glycerol, 2% SDS, 0.01% bromophenol blue] for 15 min, followed by another equilibration with 2.5% (w/v) iodeacetamide in the same buffer for 15 min. Equilibrated IPG strips were placed onto 12.5% SDS-polyacrylamide gels for the second dimensional separation. Electrophoresis gels were stained with Coomassie blue [23] and image analysis was performed with Image-Master 2D Elite Version 3.1. Two replicate 2-DE gels for each sample were used: one for Coomassie blue stain and the other for western blot analysis. To insure reproducible results, at least three spore/vegetative preparations were used for the 2D gel electrophoresis (2-DE) analysis.

#### 2-DE immunoblot assays using rabbit immune sera against *B.anthracis* live spores

Immunoblotting was conducted as described previously[24]. For western blotting, proteins on one copy 2D gels were transferred to polyvinylidene difluoride (PVDF)(Millipore,Billerica, MA, USA) membranes using transferring buffer (25 mM Trizma base, 190 mM glycine, 20% methanol) at 100 V for 90 min. After the transfer, the PVDF membrane was blocked with 5% skimmed milk prepared in PBS with 0.05% Tween 20 (PBS-T) overnight at 4°C and incubated with the anti-*B.anthracis* serum prepared above, at a dilution of 1∶2000 for 4 h at room temperature. Membranes were then washed four times with PBS-T for 20 min, developed using an Enhanced Chemiluminescence (ECL) kit (Thermo scientific, Rockford, IL, USA), and exposed to X-ray film (Kodak, Wuxi, China) for visualization of the antigenic proteins. For controls, corresponding antisera from unimmunized rabbits were collected and used for 2-DE immunoblotting. All immunogenic proteins that appeared in both the control and treated blots were excluded from the list of immunogenic candidate proteins.

#### In-gel trypsin digestion and matrix-assisted laser desorption ionization time-of-flight/time-of-flight mass spectrometry (MALDI-TOF/TOF MS)

2-DE gels and their immunoblot profiles were compared. The Coomassie-stained protein spots corresponding to the immunoreactive spots in western blotting were cut out, and in-gel protein digestion was performed as described previously [25]. Peptides from digested proteins were solubilized in 2 µl 0.5% trifluoroacetic acid. Tryptic peptides were subjected to MALDI-TOF/TOF MS. All MALDI-TOF/TOF MS measurements were performed on a Bruker UltraReflex™ III MALDI-TOF/TOF mass spectrometer (Bruker Daltonics, Karlsruhe, Germany) operated in reflection mode. Peptide mass fingerprints were analyzed and searched against the theoretical spectra of *B.anthracis* strain Ames (5311 entries, including all of the ORFs predicted on the chromosome of *B.anthracis* str. Ames; accession number NC_003997) and Sterne (5287 entries, including all of the ORFs predicted on the chromosome of *B.anthracis* str. Stern; accession number NC_005945) using the Mascot Daemon software package (Matrix Science Ltd.). The search parameters were as follows: trypsin digestion with one missed cleavage; carbamidomethyl modification of cysteine as a fixed modification and oxidation of methionine as a variable modification; peptide tolerance maximum, ±0.2 Da; MS/MS tolerance maximum, ±0.6 Da; peptide charge, 1+; monoisotopic mass. Scores >49 was significant (*p*<0.05) for a local peptide mass fingerprinting (PMF) search. For unambiguous identification of proteins, more than five peptides must be matched for a PMF search.

### Validation of immunogenicity of proteins identified by immunoproteomics

To validate the immunogenicity of the identified proteins, 12 were selected for expression and purification. On the one hand, the purified proteins were probed with the rabbit immune sera against live spores of the *B.anthracis* vaccine strain A16R. On the other hand, the purified proteins were used to immunize mice and the antisera were prepared. These immune sera against each of the 12 proteins were used to probe the whole cell lysate proteins of the A16R vaccine.

#### Proteins expression and purification

Twelve proteins were chosen for prokaryotic expression: two of which (BA0887 and BA3338) were S-layer homology domain proteins and the other 10 were all enzymes. The two categories of proteins were previously thought to be potential immunogens[26]. The primer pairs used to amplify the DNA fragments of the recombination proteins are listed in Table S1. The gene fragment was PCR amplified, restriction enzyme digested, and linked to the expression vector pET32a. The cloned gene and part of the vector fragment flanking the cloned gene were sequenced to confirm the right reading frame. The recombinant plasmid was transformed into *Escherichia coli* strain BL21. For expression of the recombinant proteins, the cells were induced by adding 1 mM IPTG when they grew to OD_600_ = 0.6–1.0, and harvested after 16 h at 16°C. The soluble recombinant proteins were identified by western blotting using the His-horseradish peroxidase (HRP) antibody and purified on a Ni^2+^ Sepharose column with gradient elution.

#### The purified proteins were probed with rabbit anti-live spore immune sera

Twelve purified proteins were electrophoresed, and the gels were then transferred to PVDF membranes (Millipore, Billerica, MA, USA) at a constant voltage of 15 V for 1 h 45 min. The transferred membranes were blocked with TBS-T (TBS with 0.05% Tween 20) containing 5% skimmed milk. The membrane was incubated for 60 min with rabbit anti-live spore immune sera (diluted to 1∶500 in TBS-T) and washed with TBST three times for 7 min. Membranes were incubated in 40 ml goat anti-rabbit IgG-HRP (Thermo Scientific, Rockford, IL, USA) regents for 60 min at room temperature and washed by TBS-T four times for 7 min. Membranes were dipped into ECL substrate solutions (Thermo Scientific) for 5 minutes and then exposed on the film (Kodak, Wuxi, China).

#### Preparation of mouse immune serum against fusion proteins

To generate polyclonal antibodies against the fusion protein, 100 µg of the purified recombination protein with Freund's incomplete adjuvant (Sigma) was injected into five female BALB/c mice by subcutaneous and intraperitoneal injection. The mice were boosted another two times at 2-week intervals with 100 µg purified protein mixed with Freund's incomplete adjuvant (Sigma). The titers of the sera were measured by indirect ELISA 7 days after the last boost, and the sera were used for immunoblotting experiments. For controls, corresponding antisera from unimmunized mice were collected.

#### Western blotting with A16R whole cell lysate proteins

About 1 ml of the vegetative cells of A16R cultured for 23 h were harvested, boiled and centrifuged and 20 µl supernatant was loaded onto 12% acrylamide gels for SDS-PAGE, and transferred to PVDF membranes (Millipore). The membranes were blocked with 5% BSA and then incubated for 1 h with the mouse control serum and mouse immune sera raised against recombinant proteins. The membranes were washed and HRP-conjugated goat anti-mouse IgG secondary antibodies (Santa Cruz Biotechnology, Santa Cruz, CA, USA) were added for 1 h. The ECL Plus Kit (Pierce, Rockford, IL, USA) was used for detection. For controls, corresponding antisera from unimmunized mice were collected and subjected to western blotting with A16R whole cell lysate proteins.

### Computational analyses of the novel identified immunogenic protein

Analyses of protein domains were carried out by searching against the Pfam and SMART databases with the online program InterPro Scan (http://expasy.org/tools). The subcellular localization predictions were carried out with the PSORTb online program (http://www.psort.org/psortb/index.html). Predictions of presence and location of signal peptides in the N-terminal 70aa of the protein were carried out with the online program SignalP (http://www.cbs.dtu.dk/services/SignalP/). Predictions of transmembrane regions and orientation of the proteins were carried out using the online program TMpred (http://www.ch.embnet.org/software/TMPRED_form.html). The functional assignments were carried out by comparing the amino acid sequences of the interest proteins with the COGs (Clusters of Orthologous Groups of proteins) database using the online program COGNITOR (http://www.ncbi.nlm.nih.gov/COG/old/xognitor.html).

## Results

### Staining and counting *B.anthracis* spores

Basic fuchsin and methylene blue staining was carried out to observe the purity of the collected spores. A large number of spores stained red were observed by light microscopy. It was concluded that the spores harvested could be used as the antigen to immunize the rabbits. The pure spores were treated with gradient dilution and plated on BHI agar for counting. The concentration of the spores was 5.5×10^9^ CFU/ml.

### Identify of immunogenic spore and vegetative proteins by immunoproteomics

**Figure 1:**
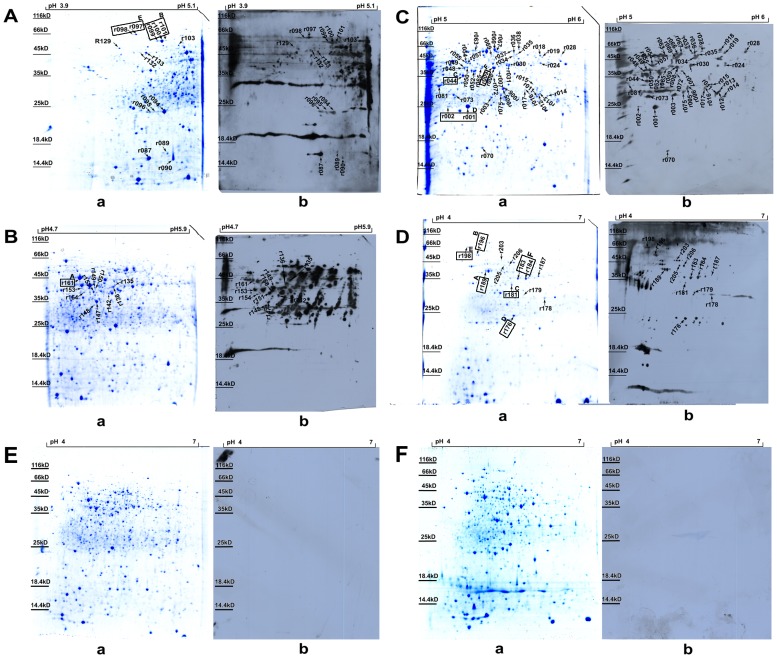
2-DE map and corresponding western blotting image of *B.anthracis* A16R vegetative and spore proteins. (A–C) Vegetative proteins for the pH 3.9–5.1, 4.7–5.9 and 5–6 strips, respectively. (D) Spore proteins for the pH 4–7 strip. (E and F) Vegetative and spore proteins for the pH 4–7 strips, respectively. (a and b) for A–D indicate 2-DE maps and the corresponding western blotting images using rabbit immune sera against *B.anthracis* live spores, respectively. (a and b) for E and F indicate 2-DE maps and the corresponding western blotting images using rabbit unimmunized sera, respectively. The spots whose names are in boxes indicate that those proteins were identified in both vegetative and spore proteins. The same upper case letter indicates the same protein in the vegetative and spore proteins.

2-DE immunoblotting was performed to investigate the immunogenic proteins using rabbit immune sera against *B.anthracis* live spores. In the 2-DE map of the vegetative proteins, 106 spots were cut and 70 spots representing 45 proteins were successfully identified by MALDI-TOF/TOF MS, while 25 protein spots were cut and 13 spots representing 11 proteins were successfully identified in the spore proteins (Fig. 1). The MALDI-TOF/TOF MS results are illustrated in the supplemental material (Tables S2 and S3). There were six proteins identified both in vegetative and spore proteins. Twenty-four proteins had been reported previously (Table 1).

**Table 1:** Immunogenic proteins identified in this study.

Vegetative immunoreactive spots	Spore immunoreactive spots	Accession No.	gene	Protein description	MW^a^	pI^b^	Note
r028	-	BA0080	-	Negative regulator of genetic competence clpC/mecB	90532	6.06	[45]
r161	r189	BA0108	tuf	elongation factor Tu	43026	4.93	[29], [41], [45]
r099\r100\r101	r196	BA0267	groEL	chaperonin GroEL	57396	4.79	[29], [41], [45]
r066\r073\r096	-	BA0309	-	1-pyrroline-5-carboxylase dehydrogenase	56418	5.43	[45]
r094\r095	-	BA0345	ahpC	alkyl hydroperoxide reductase, subunitC	20878	4.81	[45]
r012\r015\r016\r018\r030\r031\r034\r035\r037\r048\r052\r063\r075\r142	-	BA0887	eag	S-layer protein EA1	91307	5.7	[26], [27], [28], [29], [30], [31], [36], [41], [45]
r007		BA1300	potD	putrescine-binding protein	39552	5.97	[29]
r049		BA1419	ilvC-1	Ketol-acid reductoisomerase	36784	5.17	[25]
r044	r181	BA3964	tsf	translation elongation factor Ts	32529	5.25	[29], [41]
-	r187	BA4184	pdhA	Pyruvate dehydrogenase E1 component subunit alpha	41415	5.52	[41]
r001\r002	r176	BA4499	sodA-1	Superoxide dismutase	22650	5.3	[26]
r097\r098	r198	BA4539	dnaK	molecular chaperone DnaK	65784	4.65	[29], [45]
r129	-	BA4705	tiG	trigger factor	47185	4.53	[41]
r132\r133	-	BA5364	eno	enolase	46446	4.66	[37], [41], [42], [45]
r009	r183\r184	BA5369	gap-2	glyceraldehyde-3-phosphate dehydrogenase	35974	5.37	[37], [41]
r005	-	BA5565	-	hypothetical protein BA5565	22274	5.45	[25]
r081\r147	-	BA5580	fba-2	fructose bisphosphate aldolase	30825	5.06	[37]
r151	-	BA3338	-	S-layer protein, putative	40427	5.84	[26], [45]
r054	-	BA4873	ald-2	Alanine dehydrogenase	40141	5.22	[45]
-	r178	BA1021	-	hypothetical protein BALH_0915	31558	6.01	[41]
r006	-	BA5588	-	3-hydroxybutyryl-CoA dehydrogenase	31533	5.45	[41]
r024	-	BA2958	-	3-deoxy-7-phosphoheptulonate synthase	40115	5.65	[41]
r154	-	BA1998	nadE	NH(3)-dependent NAD(+) synthetase	30083	4.94	[41]
r014	-	BA5061	-	hypothetical protein	25273	5.8	[41]
-	r205\r206	BA5017	metK	S-adenosylmethionine synthetase	43464	5.2	New
-	r203	BA5147	glyS	glycyl-tRNA synthetase	53497	5.09	New
-	r179	BA1232	fabI	enoyl-(acyl carrier protein) reductase	27894	5.45	New
r153	-	BA0347	-	NH(3)-dependent NAD(+) synthetase	38454	4.99	New
r149	-	BA4861	pepQ-2	proline dipeptidase;Peptidase M24, methionine aminopeptidase	40862	5.13	New
r148	-	BA1497	resD	DNA-binding response regulator ResD;Signal transduction response regulator, receiver domain;CheY-like superfamily	27514	5.08	New
r103	-	BA0344	ahpF	alkyl hydroperoxide reductase, F subunit	55061	4.93	New
r090	-	BA2013	dpS	general stress protein;DNA-binding protein Dps;Ferritin-like protein, Dps type	16638	4.79	New
r089	-	BA5290	-	general stress protein 20U;DNA-binding protein Dps;Ferritin-like protein, Dps type	16899	4.76	New
r087	-	BA0100	rplL	50S ribosomal protein L7/L12	12510	4.71	New
r072	-	BA1555	dapB	dihydrodipicolinate reductase	29294	5.36	New
r070	-	BA1536	-	nucleoside diphosphate kinase	16590	5.24	New
r069	-	BA0346	-	5-Methylthioribose kinas	47360	5.38	New
r067	-	BA2354	mmsA-1	methylmalonic acid semialdehyde dehydrogenase	53169	5.44	New
r065	-	BA1969	thrC	threonine synthase	37723	5.32	New
r057	-	BA4274	nagA	N-acetylglucosamine-6-phosphate deacetylase	41642	5.26	New
r055	-	BA4888	ackA	acetate/propionate kinase	43234	5.25	New
r050\r135	-	BA0079	-	arginine or creatine kinase;ATP guanido phosphotransferase	40238	5.24	New
r038	-	BA4385	bfmbC	dihydrolipoamide dehydrogenase	50914	5.53	New
r036	-	BA0550	prkA	probable serine protein kinase	73341	5.65	New
r019	-	BA1511	gdhA	glutamate/phenylalanine/leucine/valine dehydrogenase	47748	5.85	New
r017\r136\r150	-	BA0509	pfl	formate acetyltransferase	84712	5.65	New
r013	-	BA2551	-	enoyl-CoA hydratase	28559	5.69	New
r011	-	BA0894	-	enoyl-CoA hydratase	28746	5.66	New
r008	-	BA4839	citZ	Citrate synthase	41928	5.36	New
r003	-	BA4194	-	2,3,4,5-tetrahydropyridine-2-carboxylate N-succinyltransferase	25745	5.42	New

Note: ^a^:Molecular Weight

b :Isoelectric Point

### Cellular function analysis of the immunogenic proteins

**Figure 2:**
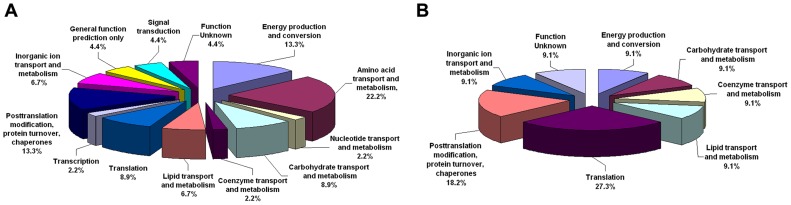
Graphic representation of the immunogenic proteins categorized according to cellular function. (A) *B.anthracis* vaccine strain A16R vegetative proteins; (B) *B.anthracis* vaccine strain A16R spore proteins.

Fig. 2 shows the cellular functions of the identified immunogenic proteins from *B.anthracis* vaccine strain A16R vegetative cells and spores. The majority of the vegetative proteins were involved in amino acid transport and metabolism, energy production and conversion, post-translational modification, protein turnover, and as chaperones (Fig. 2A). Some of the spore proteins were involved in translation and post-translational modification, protein turnover, and as chaperones (Fig. 2B).

### Validation of immunogenicity of proteins identified by immunoproteomics

**Figure 3:**
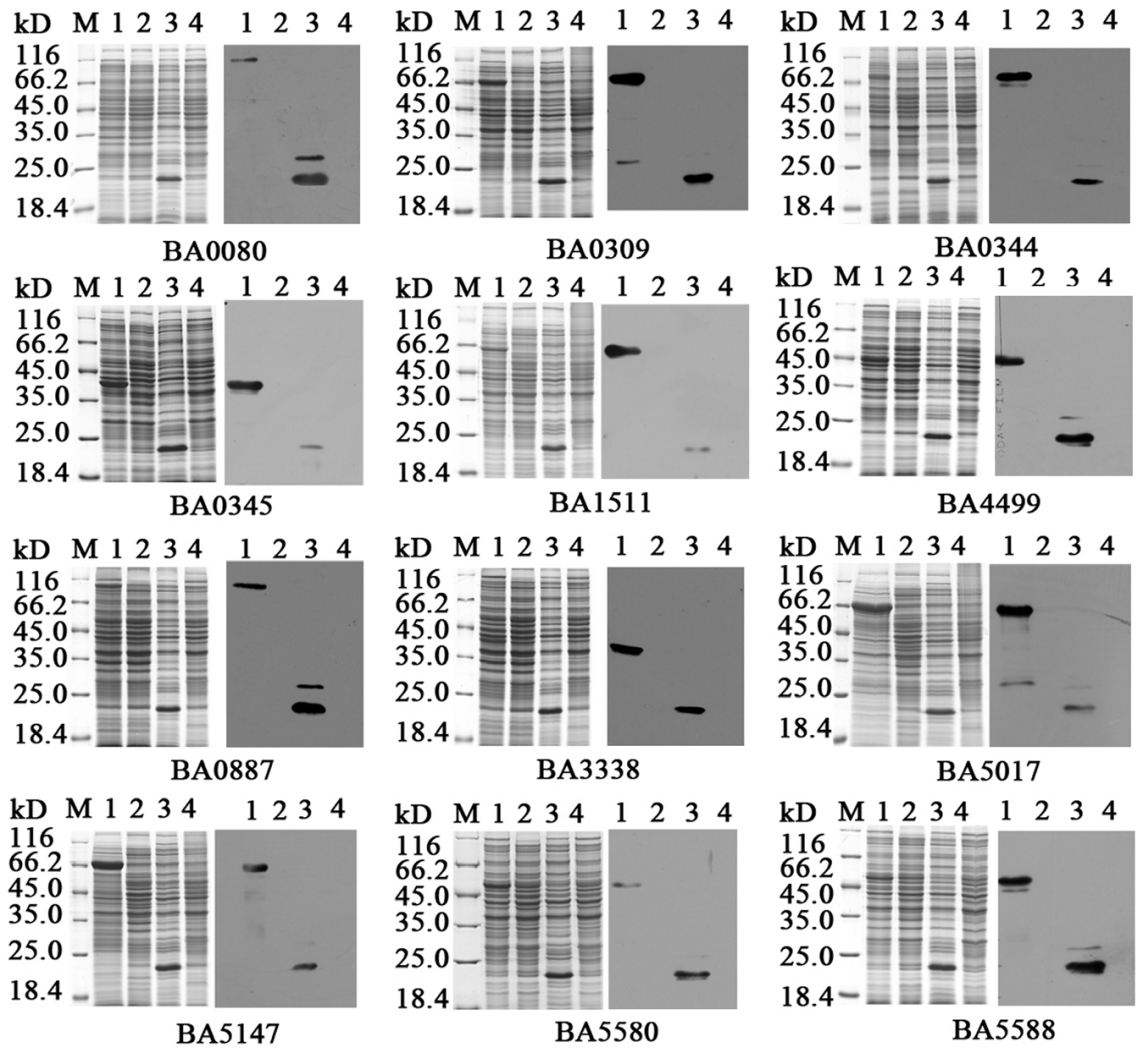
Expression of the 12 immunoproteins. The left panels are the SDS-PAGE maps and the right panels are the western blot assay with His-tag monoclonal antibody (HRP-conjugated). (1) Proteins of interest after induction; (2) proteins of interest before induction; (3) vector after induction; (4) host cell BL21. M: Unstained protein molecular weight marker (Fermentas).

To verify the immunogenicity of the candidate proteins, 12 proteins were selected for amplification and were linked to the expression plasmid pET32a. Cells were harvested before and after induction by IPTG, and the samples were separated by electrophoresis and transferred onto PVDF membranes. The His-tagged monoclonal antibody (HRP-conjugated) was used for western blotting, and the results revealed that all the expressed proteins had their corresponding theoretical molecular weight (Fig. 3). These recombination proteins were purified by Ni^2+^ Sepharose column chromatography and used as the antigen to obtain mouse polyclonal antibodies or to react with rabbit immune sera against live spores of *B.anthracis*.

**Figure 4:**
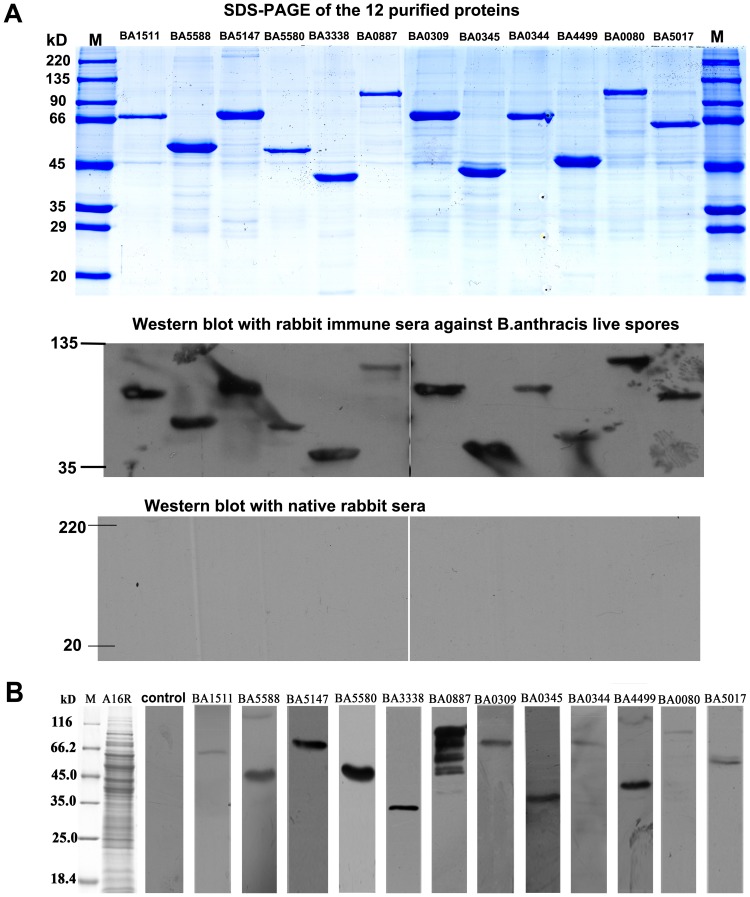
Validation the immunogenicity of the 12 proteins identified by immunoproteomics. (A) The 12 purified proteins were probed with rabbit immune sera against live spores of *B.anthracis* vaccine strain A16R. The upper panel shows the SDS-PAGE maps of the 12 purified proteins, the middle panel is the western blotting assay with rabbit immune sera against *B.anthracis* live spores and the lower panel is the western blotting assay with the unimmunized rabbit sera. M:unstained protein molecular weight marker (Cwbio, China). (B) Western blotting assays for A16R whole cell proteins using immune sera from mice vaccinated with the 12 expressed proteins. A16R: whole cell lysate proteins of *B. anthracis* vaccine strain A16R; control: A16R whole cell proteins were probed with unimmunized mice sera. M: Unstained protein molecular weight marker (Fermentas).

To confirm the immunogenicity of the identified proteins, 12 purified proteins were probed with rabbit immune serum against live spores of *B.anthracis* vaccine strain A16R. All 12 purified proteins reacted with the serum with a strong specific reactive band (Fig. 4 A).

In order to establish whether mouse polyclonal antibodies against each of the 12 recombinant proteins could react specifically with the whole cell lysate proteins of A16R, about 1 ml of the vegetative cells of A16R cultured for 23 h was harvested for SDS-PAGE. Western blot analysis was performed in which the primary antibody was the mouse immune serum against the recombinant proteins, and the second antibody was HRP-conjugated anti-mouse IgG antibody. All the mouse immune sera against recombinant proteins reacted with the lytic cell proteins of A16R with a specific reactive band, except for BA0887. The serum against protein BA0887 had several reactive bands with the lytic cell proteins of A16R, which may have been because the protein was broken into small peptides during SDS-PAGE. The results validated the immunogenicity of these proteins (Fig. 4 B).

### Computational analyses of the novel identified immunogenic protein

In this study, 26 *B.anthracis* immunogenic proteins were documented for the first time; 22 of which were enzymes, and the other four were general stress proteins, ribosomal proteins and DNA-binding response regulators. Cellular function analysis indicated that most of the novel immunogenic proteins were involved in amino acid transport and metabolism and energy production and conversion. The subcellular localization analysis indicated that two (BA1213 and BA0344) of the 26 proteins were cytoplasmic membrane proteins and the others were cytoplasmic proteins. Predictions of transmembrane regions with the TMpred program indicated that 16 proteins had transmembrane regions and the BA0344 protein had three transmembrane regions. Signal peptide predictions indicated that no signal peptides were present in the N-terminal 70aa of the 26 proteins (Table S4).

## Discussion

The aim of this study was to identify the immunogenic proteins of *B.anthracis* spore and vegetative forms as candidates for the development of vaccines and inhibitors against anthrax. Spores are the dormant state of *B.anthracis* and their germination and early outgrowth are considered as targets against anthrax. Prevention of *B.anthracis* at the onset of its life cycle could suppress downstream events such as bacteremia and toxemia, which are associated with disease manifestations. Therefore, the spores, which were not only the infective form of *B.anthracis* but also the antigens that elicited a host immune response, were chosen for investigation in the present study.

Among the identified proteins, EA1, which is encoded by the *eag* gene and has been reported several times before, was found only in the vegetative proteins in the present study. EA1 protein belongs to the S-layer proteins with various functions, including shape maintenance, host evasion, cell adhesion, and resistance to phagocytosis [26], [27], [28], [29]. EA1 protein contains a standard signal peptide and is considered to be a major surface antigen and vaccine carrier *in vivo* [30], [31], [32]. Apart from the two predominant S-layer proteins (EA1 and Sap) on pXO1 previously identified by Okinaka[33], Mock and Fouet were the first to report that the *B.anthracis* genome harbors additional genes coding for S-layer homology (SLH) proteins that may constitute potential vaccine candidates[34], [35]. Computational analysis of the *B.anthracis* draft version of the chromosome for ORFs containing at least one SLH domain has revealed the presence of 20 putative S-layer homology domain proteins[26]. In this study, the identified immunogenic proteins list also included a novel putative S-layer protein BA3338, which consisted of an N-terminal signal peptide for export, and three S-layer homology domains for anchorage to the cell wall. BA3338 is highly similar to internalin of *Bacillus cereus*, which is considered to be a candidate virulence factor [36].

Recently, to understand the pathogenicity and immunogenicity of *Streptococcus iniae*, Shin [37] identified that GAPDH, fructose bisphosphate and enolase have high immunogenicity. It has been reported that GAPDH (BA5369) can bind various mammalian proteins, such as lysozyme, fibronectin and cytoskeletal proteins myosin and actin[38], [39], [40]. Therefore, it can be concluded that GAPDH plays an important role in adhesion to host cells and their colonization. In our study, GAPDH protein was found in both the spore and vegetative proteins of *B. anthracis* A16R. This is consistent with the study of Delvecchio who has concluded that GAPDH is involved in spore germination, energy metabolism and strain growth[41]. Enolase (BA5364) has been previously identified as a component of Anthrax Vaccine Adsorbed and has a role in the virulence and protection of *B.anthracis* [42]. The E1 component subunit α (BA4184) of the pyruvate dehydrogenase (PDH) complex, in which another two proteins are dihydrolipoyl transacetylase (E2) and dihydrolipoyl dehydrogenase (E3), takes part in the tricarboxylic acid cycle to generate NADH, ATP, and reduced FAD [41], [43] Although its function in *B.anthracis* has not been reported, the PDH complex has been shown to be highly immunogenic in other bacterial species, such as *Mycoplasma capricolum*, *Neisseria meningitidis*, and *Mycoplasma hyopneumoniae*. Recently, PDH has been tested as a DNA vaccine against *Mycoplasma mycoides subsp. mycoides*, the causal agent of contagious bovine pleuropneumonia [43].

Translation elongation factors (Tuf and Tsf) and chaperones (DnaK and GroEL) are important core metabolic proteins that facilitate rapid growth upon germination. Additionally, alkyl hydroperoxide reductase, subunit C (BA0345), has been reported to be immunogenic in the *B.anthracis* membrane fraction [44].

In summary, the present study investigated the immunogenic proteins of the spore and vegetative forms of *B.anthracis* vaccine strain A16R using 2-DE immunoblot assays. Forty-five and 11 immunogenic proteins were found in the vegetative and spore forms, respectively. Fifty immunogenic proteins were identified. Twenty-four of these have been reported previously and the other 26 were new immunogenic proteins found only in this study. Seven common antigenic proteins were observed on both 2-DE immunoblot profiles, whereas 38 and four proteins, respectively, were found as specific antigens for vegetative and spore forms of *B.anthracis*. Twelve proteins were selected for prokaryotic expression and antisera were made to validate their immunogenicity further. In fact, some of these immunogenic proteins might be used as novel vaccine candidates themselves, or inclusion of one or more of these newly identified immunogenic proteins in a PA-base vaccine may serve to enhance protective efficacy. Future work probing the identified immunogenic proteins with the serum from convalescent anthrax patients, and *in vivo* protection experiments, should be carried out to validate the immunogenicity and protective efficacy. The immunoproteomics approach is valuable for identifying immunogenic proteins for use in vaccine development.

## Supporting Information

Table S1 **12 pairs of primers.**
(XLS)

Table S2 **Information on the immunogenic vegetative cell proteins.**
(XLS)

Table S3 **Information on the immunogenic spore proteins.**
(XLS)

Table S4 **The computational analyses of the novel immunogenic proteins identified in this study.**
(XLS)
